# Spontaneous duodenocutaneous fistula: a rare presentation of perforated duodenal ulcer

**DOI:** 10.1515/iss-2023-0051

**Published:** 2024-04-09

**Authors:** Isabel Barreto, Arnold Kohler, René Fahrner

**Affiliations:** Department of Surgery, 30857 Bürgerspital Solothurn, CH-4500 Solothurn, Switzerland; Department of Surgery, University Hospital Zürich, University of Zürich, Zürich, Switzerland; Jungfraupraxis Interlaken, Interlaken, Switzerland; Department of Vascular Surgery, University Hospital Bern, University of Bern, Bern, Switzerland

**Keywords:** duodenal ulcer, duodenocutaneous fistula, non-surgical treatment, nutrition, complication

## Abstract

**Objectives:**

Fistula formation between the duodenum and the skin of the anterior abdominal wall is a rare complication and reported most often following surgery. To the best of our knowledge, the development of a spontaneous duodenocutaneous fistula in association with duodenal ulcer has only been reported once.

**Case presentation:**

A 52-year-old female patient presented at the emergency department with a painful ulcer and erythema on the right abdominal wall. On admission, she was in extremely poor general and nutritional condition. Laboratory analysis revealed inflammation. An empiric antibiotic therapy was initiated; parenteral nutrition, fluid, and electrolyte resuscitation were started. An enterocutaneous fistula was postulated and confirmed by endoscopy identifying a perforated duodenal ulcer. Surgery was not a valuable option and a Foley catheter was inserted through the fistula. During further endoscopic interventions, the Foley catheter was first replaced by a jejunal tube and later by a percutaneous endoscopic gastrostomy with a jejunal limb for enteral nutrition. The fistula output decreased, the local infection was controlled and the nutritional status improved.

**Conclusions:**

Three months later the fistula was closed and the gastrostomy tube was removed. After 2 years the patient was in good general and nutritional condition.

## Introduction

Enterocutaneous fistula is defined as a connection between the lumen of the gastrointestinal tract and the skin. In the majority of cases they are caused by surgery, usually as a result of bowel anastomotic leak or missed enterotomy [1]. A minority of enterocutaneous fistulas arise spontaneously, most commonly due to Crohn’s disease. Other etiologies of spontaneous enteric fistula formation and persistence include foreign body, radiation, inflammation and infections (tuberculosis, actinomycosis), neoplasia and distal intestinal obstruction [1].

The pathogenesis of peptic duodenal ulcer is often induced by an imbalance of defensive (mucus layer, prostaglandins, mucosal blood supply, cellular regeneration) and aggravating (bile salts, increased acid, pepsin, ethanol, drugs) factors [2]. Particularly, non-steroidal anti-inflammatory drugs (NSAIDs) and *Helicobacter pylori* infections are responsible for the majority of peptic ulcers. However, there are peptic ulcers even in the absence of *H. pylori* infection and NSAID treatment, such as idiopathic ulcers, Cushing’s ulcer, in case of Zollinger-Ellison syndrome and after abdominal radiotherapy [2]. Most duodenal fistulas are reported in Crohn’s disease, pancreatitis, after surgical interventions or trauma, and seldom in duodenal ulcer or other underlying disease [3], [4], [5]. The basis of medical treatment of peptic ulcer includes the reduction of acid levels in the upper gastrointestinal tract, eradication of *H. pylori* and prevention of external toxic substances [2]. In case of perforated ulcers, surgery is the primary treatment modality, whereas conservative treatment regimens have been published as well [2]. Depending on the clinical condition of the patient, cause and location of the fistula and accompanying complications, there are several treatment options varying from multimodal non-operative treatment (drainage, clip, parenteral nutrition, octreotide) to surgery, including resections, direct closure (with or without omentoplasty) and negative pressure therapy [2, 4], [5], [6], [7], [8].

Spontaneous duodenocutaneous fistula is a potential complication of duodenal ulcer. Nevertheless, to the best of our knowledge, only one case has been published in literature so far [9]. We report here the second case of spontaneous duodenocutaneous fistula because of duodenal ulcer with successful non-surgical management.

## Case presentation

**Figure 1:**
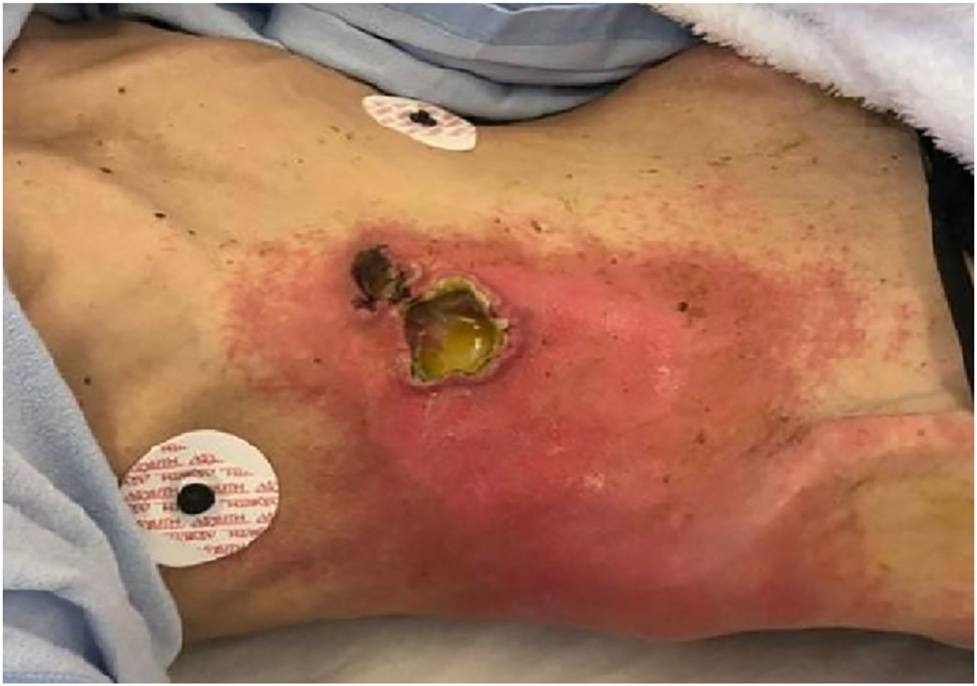
Abscess formation on the right anterior abdominal wall due to duodenocutaneous fistula.

A 52-year-old female patient presented at the emergency department with a painful ulcer and erythema on the right anterior abdominal wall. She reported no previous surgeries, but paranoid schizophrenia and anorexia, without regular medication. Because of the abdominal pain she lost 10 kg of weight during the previous weeks and presented with an initial weight of 35 kg and 168 cm height (body mass index 12.5 kg/m^2^). On admission at the emergency department she was in extremely poor general and nutritional condition. Furthermore, she was hypotonic, hypothermic and the physical examination revealed a 5×5 cm ulcer with abscess on the right upper anterior abdominal wall with surrounding erythema (Figure 1).

Laboratory analysis revealed inflammation (leukocytes 12.5 G/l, C-reactive protein 146 mg/L) so that an empiric antibiotic therapy (piperacillin/tazobactam and clindamycin), fluid and electrolyte resuscitation were initiated. A high-dose proton pump inhibitor (PPI) therapy was also administered. Because of severe malnutrition with an albumin level of 12 g/L, total parenteral nutrition was established with initial supplementation of 1100 kcal, which was increased up to 1600 kcal intravenousely. Under psychopharmacological treatment the psychotic symptoms were continuously improved.

**Figure 2:**
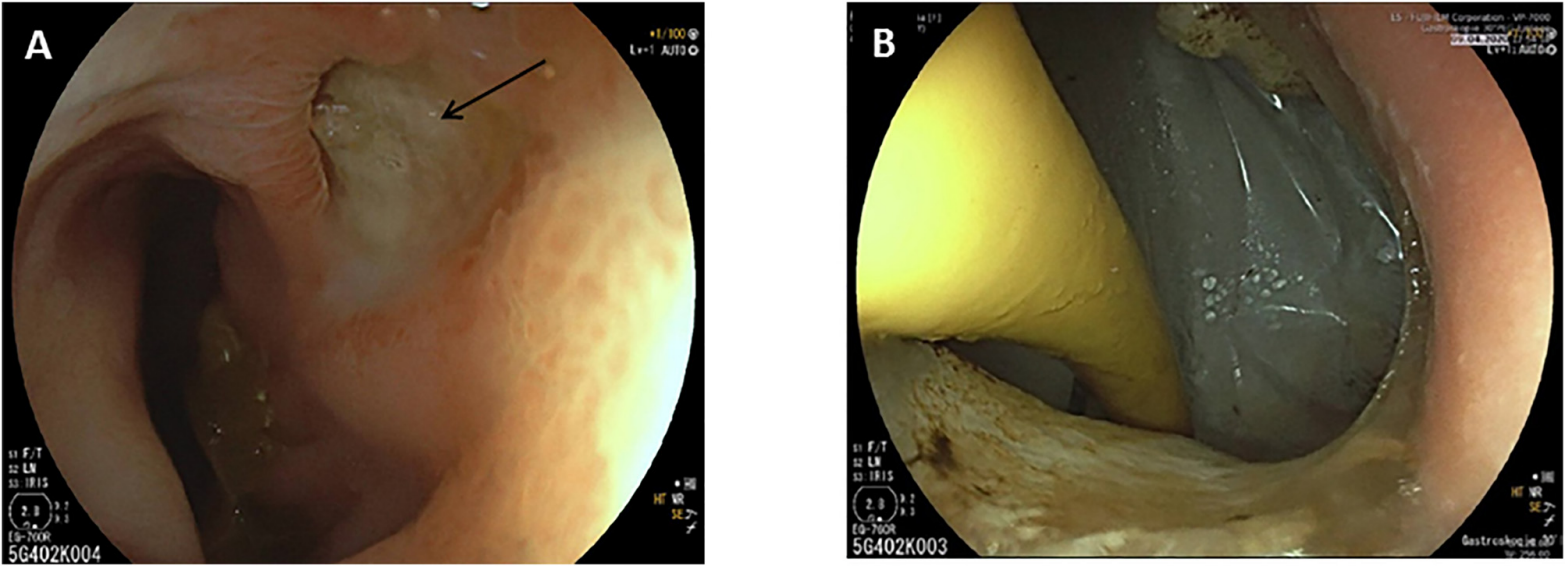
Endoscopic image showing the duodenal ulcer (A, fistula opening marked by an arrow) and the inserted Foley catheter through the fistula opening (B).

**Figure 3:**
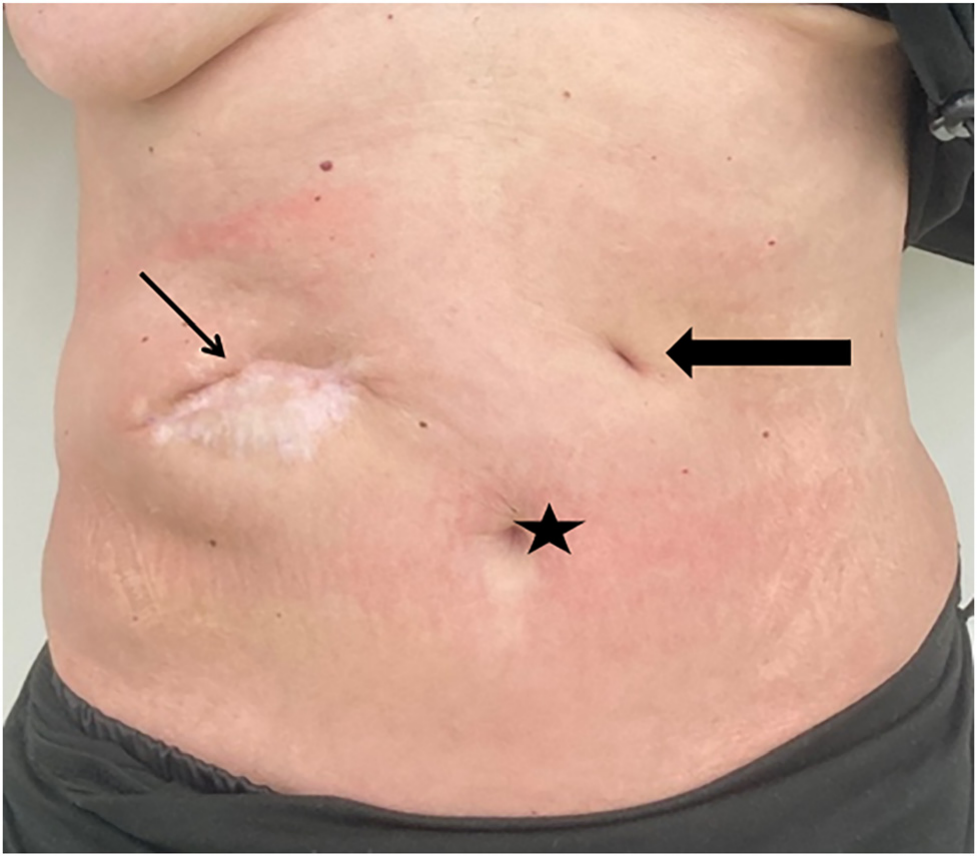
Abdominal situation about two years after the initial presentation showing proper secondary healing of the abscess formation and unremarkable scar of the gastrostomy tube site. As secondary finding the patient gained weight and was in good clinical condition. Star – umbilicus; thin arrow – secondary healed abscess formation; bold arrow – scar of the gastrostomy tube.

Owing to stool evacuation through the abdominal ulcer, an enterocutaneous fistula was postulated. An endoscopy identified a perforated duodenal ulcer (Figure 2A). As surgery was not a valuable option due to the poor general condition, a Foley catheter was inserted through the fistula to obtain a controlled drainage (Figure 2B). During further endoscopic interventions, the Foley catheter was first replaced by a jejunal tube and later by a percutaneous endoscopic gastrostomy with a jejunal limb for enteral nutrition (enteral supplementation of 1250 kcal), so that the parenteral supplementation could be reduced. These interventions led to decreased fistula output. The local infection was controlled by vacuum therapy and the nutritional status improved (albumin 22 g/L and weight of 40.5 kg at discharge), so that the patient could be discharged to a care facility with full oral alimentation and low dose enteral supplementation via the gastrostomy tube. Three months later the fistula was closed and the gastrostomy tube was removed. After 2 years the patient was in good general and nutritional condition (weight about 50 kg; Figure 3). The ulcer was completely closed and enteral nutrition was possible without obstruction. The patient gave written informed consent to publish her medical records and images.

## Discussion

We report a rare complication of a spontaneous perforated duodenal ulcer owing to duodenocutaneous fistula formation, which was successfully treated by non-operative treatment and interdisciplinary management. The patient initially presented in poor general condition with extreme malnutrition and cachexia, so that she did not qualify for a surgical approach and multimodal non-operative treatment was installed in our surgical department. The stage-dependent treatment was adapted, and the general condition of the patient improved. The surgical treatment was continuously evaluated but, due to the favorable course the non-operative management, the latter could be continued until the complete healing of the fistula and cutaneous ulcer.

Comparable to the reported case, *H. pylori* is known to be one of the major causes of peptic duodenal ulcer [2]. An early diagnosis and eradication of this germ avoids severe duodenal ulcer formation and perforation. In the presented case no previous abdominal pain was reported, probably due to the psychotic disorder and malnutrition with cachexia and abdominal symptoms. Even the cutaneous alteration was misinterpreted by the patient and medical support was prevented until the situation exacerbated and the general condition became disastrous. During the hospitalization other common causes of duodenal ulcer were ruled out.

An abdominal computed tomography with intravenous and oral contrast may delineate the anatomy of the fistula, but also demonstrate intraabdominal abscesses, fluid collections and potential intestinal obstruction. In our case, however, the abdominal computed tomography was not conclusive due to the cachexia. Finally, an endoscopy showed the duodenal ulcer and fistula opening as source for the cutaneous lesion and stool evacuation. Other diagnostic tools have been reported such as fistulography or upper gastrointestinal X-ray studies to demonstrate the duodenal perforation and fistula [3, 9].

First line therapy for most of the uncomplicated duodenal ulcer is medical treatment with PPI and eradication of *H. pylori* [2]. Surgery is only needed in case of complications such as bleeding or perforation. The acute management of enterocutaneous fistulas focuses on fluid and electrolyte resuscitation, including total parenteral nutrition, as well as further treatment of infection and sepsis. Fistula-associated abdominal sepsis (e.g., intra-abdominal abscess, peritonitis, or subcutaneous infection) needs to be recognized and promptly treated with antibiotics, percutaneous catheter drainage, and/or operative drainage for source control. In our case the initial general condition of the patient was massively impaired, so that a primary surgical approach despite perforation and fistula was not possible and fluid/electrolyte resuscitation and sepsis control was incipiently the goal. As first interventional step a Foley catheter was introduced through the fistula into the gastrointestinal lumen, a previously reported procedure to get local infectious control [3]. It has been shown that total parenteral nutrition is associated with a reduced fistula output and therefore, depending on the etiology of the fistula, enables a spontaneous closure [3]. In our case, after initial stabilization and definite diagnosis of a perforated duodenal ulcer, percutaneous endoscopic gastrostomy with a jejunal limb for physiological enteral nutrition was inserted, allowing a bridging and exclusion of the fistula defect. Owing to these non-surgical interventions the fistula closed and the recovery of the patient was favorable.

In conclusion, spontaneous duodenocutaneous fistula caused by a perforated duodenal ulcer has been rarely reported in the literature [9]. More frequently, enterocutaneous fistulas are reported as complications after visceral or kidney surgery, as well as in pancreatitis, trauma or inflammatory bowel disease [3, 4, 6, 8]. Leading factors for mortality of these patients were reported as localization of the fistula and etiology, higher volume fistula output, existing malnutrition, uncontrolled sepsis, and delay of diagnosis [3, 10]. Dependent on the clinical condition of the patient, cause of fistula and accompanying complications, there are several treatment options varying from multimodal non-operative treatment (drainage, clip) to surgery, including resections especially in case of complications (e.g., bleeding) or unsuccessful non-operative treatment. Therefore an interdisciplinary management is necessary to provide the optimal treatment depending on the patient’s condition. We report a successful non-operative treatment of a rare spontaneously perforated duodenal ulcer with development of a duodenocutaneous fistula.

## Supplementary Material

Supplementary Material
