# Supplementary material for: Spontaneous duodenocutaneous fistula: a rare presentation of perforated duodenal ulcer
Source: Innov Surg Sci. 2024 Apr 9;9(2):109–12. doi: 10.1515/iss-2023-0051 (PMC11294516; doi:10.1515/iss-2023-0051)
Supplement: Supplementary file 1 — Supplementary Material [file j_iss-2023-0051_suppl_001.pdf]

Reviewer Assessment

R. Fahrner, I. Barreto & A. Kohler: Spontaneous duodenocutaneous fistula: a rare presentation of perforated duodenal ulcer

Reviewers' Comments to Original Submission

Reviewer 1: anonymous

Date received: 18-Oct-2023
Reviewer recommendation: **Accept in present form**
Reviewer overall scoring: **High**

Comments to author:
The author published an interesting case report of a rare complication in duodenal ulcer with successful non-operative treatment. The management of difficult intestinal fistulas regardless of any cause should be interdisciplinary and include different treatment options considering patients condition.

Reviewer 2: Franzke, Timm

Date received: 26-Oct-2023
Reviewer recommendation: **Return to author for minor modifications**
Reviewer overall scoring: **Medium**

Comments to author:
Please state the patient's height and weight as well as BMI. How was the albumin in progress. How many calories did they start with and what diet was the patient discharged with? What was the weight curve like?

Authors' Response to Reviewer Comments

Date received: 29-Oct-2023

Reviewer 1:

Thank you for your comment. We inserted passages reflecting this interdisciplinary issue (discussion 1st and last paragraph).

Reviewer 2:

The Thank you this comment.

We inserted a statement regarding the patient's height, weight and BMI in the results section (1st paragraph).
The minimal albumin level was 12 g/l and reached a maximum of 22 g/l at discharge from the hospital. We started with 1100 kcal intravenously, which were increased up to 1600 kcal after a few days with regular lab controls for re-feeding syndrome. After establishment of the jejunal feeding tube, the enteral supplementation was performed with 1200 kcal and the iv treatment was reduced. At discharge the patient obtained full oral intake with an additional treatment via the jejunal tube. We included the data into the results section.

We included the weights on admission, discharged and control after 2 years in the results section.

Comments by the Editorial Office to the Editor-in-Chief Decision

All reviewer comments were addressed adequately and the manuscript should be published in its present stage.
